# Potential of selected plant extracts to control severe subacute ruminal acidosis in vitro as compared with monensin

**DOI:** 10.1186/s12917-022-03457-4

**Published:** 2022-09-24

**Authors:** Mariam G. Ahmed, Adham A. Al-Sagheer, Samir Z. El-Zarkouny, Eman A. Elwakeel

**Affiliations:** 1 Department of Animal and Fish Production, Faculty of Agriculture (El-Shatby), Alexandria University, Alexandria, 21545 Egypt; 2 Animal Production Department, Faculty of Agriculture, Zagazig University, P.O. Box. 44511, Zagazig, Egypt

**Keywords:** Ruminal acidosis, Plant extracts, Cinnamon extract, Monensin

## Abstract

**Background:**

In recent years, researchers have become increasingly interested in developing natural feed additives that can stabilize ruminal pH and thus prevent or eliminate the risk of severe subacute rumen acidosis. Herein, 3 experiments were conducted using a semi-automated in vitro gas production technique. In the experiment (Exp.) 1, the efficacy of 9 plant extracts (1.5 mg/ml), compared to monensin (MON; 12 μg/ml), to counteract ruminal acidosis stimulated by adding glucose (0.1 g/ml) as a fermentable carbohydrate without buffer was assessed for 6 h. In Exp. 2, cinnamon extract (CIN) and MON were evaluated to combat glucose-induced acidosis with buffer use for 24 h. In Exp. 3, the effect of CIN and MON on preventing acidosis when corn or barley grains were used as substrate was examined.

**Results:**

In Exp. 1, cinnamon, grape seeds, orange, pomegranate peels, propolis, and guava extracts significantly increased (*P* < 0.05) pH compared to control (CON). Both CIN and MON significantly increased the pH (*P* < 0.001) but reduced cumulated gas production (*P* < 0.01) compared to the other treatments. In Exp. 2, the addition of CIN extract increased (*P* < 0.01) pH value compared to CON at the first 6 h of incubation. However, no significant differences in pH values between CIN and CON at 24 h of incubation were observed. The addition of CIN extract and MON decreased (*P* < 0.001) lactic acid concentration and TVFA compared to CON at 24 h. The CIN significantly (*P* < 0.01) increased acetate: propionate ratio while MON reduced it. In Exp. 3, both CIN and MON significantly increased (*P* < 0.05) ruminal pH at 6 and 24 h and reduced lactic acid concentration at 24 h compared to CON with corn as substrate. However, CIN had no effect on pH with barley substrate at all incubation times.

**Conclusions:**

It can be concluded that CIN can be used effectively as an alternative antibiotic to MON to control ruminal acidosis when corn is used as a basal diet.

## Background

Ruminal acidosis is a prevalent digestive disorder in beef and dairy cattle, particularly during transition periods [1]. These animals depend on consuming high content of fermentable carbohydrates to improve their milk and meat productivity. These acidogenic diets increase the accumulation of organic acids upon fermentation by rumen microflora within the rumen [2]. The increased production of ruminal organic acids without sufficient neutralization results in a decline in pH values [3]. Introducing larger amounts of fermentable carbohydrates too quickly in the diets of ruminants promotes uncontrolled growth of lactate-producing bacteria and excessive amounts of lactate, resulting in decreased ruminal pH further below 5.0 (acute), inhibiting microbial fermentation [4]. In particular, subacute ruminal acidosis has a high incidence (19–26%) in early and mid-lactation dairy cows [5]. The consequences of severe subacute ruminal acidosis comprise milk fat depression, loss of appetite, low fiber digestion, liver abscesses, inflammatory reactions, diarrhea, and increased bacterial endotoxin release [6]. Changes in ruminal microbial populations and diet type have been linked to subacute ruminal acidosis [7]. For instance, protozoa numbers begin to decline, followed by a further decrease in Gram-negative bacterial numbers, while Gram-positive bacterial numbers rise [8, 9].

Several approaches used to control ruminal acidosis disorder involve using feed additives such as ionophores. Monensin (MON) is the most common ionophore used in ruminant diets that improve ruminal microbial fermentation [10]. Also, it manipulates the organic acids production by decreasing lactate production [11] and increasing propionate production [2]. However, the European Union banned the use of antibiotics in ruminant diets in January 2006 because of its residues in milk and meat, which are associated with adverse effects on human health [12].

Recently, several researchers have focused on using botanical compounds as potential and safe alternatives for antibiotics in ruminant diets. Botanical extracts are herbal plants with high content of plant secondary compounds (PSCs) such as saponin, essential oils, and phenolic compounds (e.g., tannins and flavonoids) [13, 14]. These components have many properties, such as anticarcinogenic, anti-inflammatory, antioxidant, and antimicrobial [15]. Many studies have reported that these PSCs enhance rumen fermentation characteristics by reducing methane emission and increasing animal productivity [16–18]. For instance, Wall, et al. [19] reported that a blend of plant extracts containing cinnamaldehyde, the main bioactive of cinnamon, can increase milk production and dry matter intake in lactating dairy cows. Also, cinnamaldehyde-containing mixture supplementation of lactating dairy cows at a moderate dose (640 mg/d) increased the milk fat and protein content [20].

However, the influence of plant extracts and PSCs on rumen acidosis is still scarce, and some studies noticed that lactic production bacteria were inhibited by the use of ethanolic extracts of Australian plants and essential oils [21, 22]. The supplementation of flavonoid extracts blends effectively prevented the reduction of pH and enhanced the rumen microbial population by modulating lactate-consuming bacteria in steers fed high concentrate diets [23]. In the present study, we have chosen 9 plant extracts from different origins based on their high content of phenolic compounds [24].

We hypothesized that these plant extracts would control lactic acid production in the rumen by stimulating lactic acid utilizing bacteria and inhibiting lactic acid-producing bacteria, same as MON. Thus, this study aimed to initially screen nine plant extracts against in vitro rumen acidosis. (ii) Then, evaluating the extent of protection against acute and sub-acute acidosis by selecting the promising plant extracts from the initial screening, with effects similar to MON. (iii) Evaluating selected plant extracts on the degradation of different ingredients (barley and corn grains) in vitro.

## Methods

The current research was performed at the Animal Nutrition Laboratory of Animal and Fish Production Department, Faculty of Agriculture (El-Shatby), Alexandria University.

### Plant materials and extraction

Nine plant extracts were used in the present study: Agricultural by-products of pomegranate peels (*Punica granatum*)**,** orange peels (*Citrus sinensis*), and grape seed (*Vitis vinifera*) were obtained from a food factory in the industrial region of Borg EL-Arab, Alexandria. Tree leaves of olive (*Olea europaea*), guava (*Psidium guajava*), and mango (*Mangifera indica*) were collected from a private orchard in Alexandria, and green tea leaves (*Camellia Sinensis*) from Abosheba Company, Jeddah, Saudi Arabia. Propolis powder was supplied by a trading Company Henan, China, and the grounded cinnamon (*Cinnamonum cassia*) was purchased from Royal Spices Company (Dong Nai, Vietnam).

Leaves and agricultural by-products were dried at 50 °C in a forced oven for 72 h, then ground to pass a 1 mm screen. Ten grams of powdered plant material were extracted in ethanol (80:20, v/v) at a temperature of 50 °C as described by Zarina and Tan (2013). Extracts were filtered by Whitman filter paper No, 42 (125 mm) and evaporated under pressure at 40 °C using rotary evaporation (Jobling laboratory Division, UK). Extracts were freeze-dried and stored at 4 °C for later assay. The contents of plant extracts of total phenols (TPs), total tannins (TTs), condensed tannin (CT), and total flavonoids (TFs) were determined calorimetrically (Table 1). Total phenols were estimated using Folin-Ciocalteau reagent by the procedure explained by Makkar, et al. [25]. Total tannins (TTs) were calculated by differences between TPs before and after precipitated tannins by polyvinyl poly pyrrolidone (PVPP) and the results were expressed as tannic acid equivalent [26]. Condensed tannin was determined using HCl-butanol reagent, and leucocyanidin was used as standard [27]. Total flavonoids were assayed using the aluminum chloride colorimetric method according to Zarina and Tan [28].

**Table 1:** Chemical analysis of plant extracts for phenolic compounds

Ethanolic extracts	mg/g dry matter of extracts			
	T. phenols	T. tannins	C. tannins	T. flavonoids
*Agriculture by products*				
Pomegranate peels	135.09	103.39	0.20	0.45
Orange peels	28.41	4.78	0.04	0.07
Grape seeds	336.52	92.48	16.16	3.60
*Leaves*				
Olive	65.90	23.24	0.21	0.82
Guava	306.59	143.11	0.76	1.75
Green tea	270.00	237.97	2.26	1.15
Mango	141.52	109.17	0.21	0.75
*Others*				
Propolis	93.26	41.33	0.28	0.83
Cinnamon	181.71	65.96	28.06	3.00

*T. phenols* Total phenols (eq. mg tannic acid/g), *T. tannins* Total tannins (eq. mg tannic acid/g), *C. tannins* Condensed tannin (eq. mg leucocyanidin/g), *T. flavonoids* Total flavonoids (eq. mg rutin/g)

### Animal donor and inoculum preparation

Rumen fluid was collected from three slaughtered Egyptian buffalo heifers (450 ± 50 kg, body weight) in each experiment at an abattoir belonging to the Faculty of Agriculture (El-Shatby), Alexandria University, Alexandria, Egypt. Collection of ruminal content from slaughtered animals saves money and overcomes the need for surgical cannula in live animals with complete adherence to animal protection law [29]. Also, the use of rumen fluid from slaughtered animals has been suggested and documented as an alternative in several earlier in vitro fermentation studies [30–32]. The slaughtered animals were fed on a conventional feed for meat production, which contained 70% of concentrate mixture (16% CP) and 30% of Berseem hay. The rumen was cut open with a knife after 15 minutes of slaughtering, and the contents were taken from various positions within the rumen. The rumen content was immediately strained through four layers of cheesecloth, then placed in pre-warmed thermo-containers to keep its temperature at 39 °C and under anaerobic conditions, and then transported directly to the laboratory. Rumen fluid was again strained through four layers of cheesecloth and mixed before incubation. The initial pH of rumen fluid was measured using a portable pH meter (GLP21 model; CRISON, Barcelona, Spain) in each experiment at the laboratory.

### Experiment 1, initial screening

#### Experimental design, treatments, and incubation system

Nine plant extracts compared to MON were evaluated against ruminal acidosis using the procedure described by Hutton, et al. [33]. A semi-automated in vitro gas production technique was used with a pressure transducer and data recorder (GN200, Sao Paulo, Brazil). The difference in pH and gas production values between treatments during incubation is used as an indicator of the effect of MON and plant extracts on protection against acidosis. The incubation period was 6 h without using buffer and substrate. Acidosis was stimulated by adding glucose as a fermentable carbohydrates source without using a buffer for two reasons: i) to assess differences in the pH value among treatments ii) to avoid the delay in the drop of pH readings. The treatments were control (without supplementation), MON, pomegranate peels extract**,** orange peels extract, grape seed extract, olive leaf extract, guava leaf extract, mango leaf extract, green tea leaf extract, propolis extract, and cinnamon extract (CIN). The tested concentration of the plant extracts has been selected based on a preliminary experiment (data not shown). In the earlier experiment, we have tested the 9 plant extract at a concentration of 1 mg/ml following the study of Durmic, et al. [34]. However, we did not notice any significant differences among the treatments. Hence, we chose a higher concentration (1.5 mg/ mL) to be assessed. Sodium monensin (Rumensin®, Elanco, Itapira, Brazil) was used as a positive control at 12 μg/ml, according to Ala, et al. [35]. Plant extracts and MON were dissolved in dimethyl sulphoxide (SDFCL sd fiNE-CHEM limiTEd St. Mumbai, India). Based on our laboratory protocol, 0.45 g of alfalfa hay and 4.5 g of glucose were weighed into serum bottles (120 ml) and were used as a negative control. Then it was incubated at 39 °C.

A total of 45 mL of the ruminal inoculum was placed in the 120-mL serum bottles and then dissolved plant extracts were added at 450 μl per 45 ml of rumen fluid. The initial pH of mixed rumen fluid was measured. Dimethyl sulfoxide was added at 450 μl of solvent to the control bottles. All bottles were gassed with CO_2_ and sealed with a rubber stopper and aluminum crimps. The pressure in the bottle’s headspace was adjusted to zero by inserting a 23 G needle in the stopper of the bottles, then all bottles were transferred to the incubator and incubated at 39 °C for 6 h.

#### Sample collection

The incubated bottles were removed from the incubator after 2, 4, and 6 h to measure gas pressure by inserting a 23 G needle in the headspace using a pressure transducer and data logger (GN200, Sao Paulo, Brazil) according to Mauricio, et al. [36]. The bottles were kept in a water bath at 39 °C during the recordings of pressure gas to avoid errors in the pressure readings [37]. After each reading, the 23 G needle was inserted in the rubber stopper of the bottles to release gas pressure as excessive pressure gas (48.3Kpa) may negatively influence microbial growth [38]. At the end of the incubation period (6 h), the rubber stoppers were removed, and the pH values were measured.

### Experiment 2

#### Experimental design, treatments, and incubation conditions

This experiment assessed the extended protection against sub-acute and acute acidosis for CIN compared to MON by measuring VFA and lactic acid production. The incubation conditions of the second experiment were similar to the first experiment with the following differences: fermentation time was increased from 6 to 24 h. Buffer was used in the assay because the pH value may decline due to the accumulation of organic acids over 24 h. The incubation media (45 ml) was bottled consisting of 22.5 ml of ruminal fluid and 22.5 ml of McDougall^’^s buffer [39]. The glucose concentrations (acidosis stimulant), CIN, and MON were the same as in the first experiment. The treatments were control (without any supplementation), CIN, and MON.

#### Sample collection

Cumulative gas production was measured after 2, 4, 6, 9, 12, and 24 h of incubation. Carefully, 1 ml of liquid phase was taken by inserting 23 G needle in rubber stopper of the bottle after 6, 12, and 24 h of incubation to estimate pH value. At the end of incubation time (24 h), 1 ml of liquid phase was transferred into 1.5 ml Eppendorf tube, then 200 μL of meta-phosphoric acid 25% (w/v) was added and stored at − 20 °C for later determination of VFA concentration according to the methods described by Palmquist and Conrad [40]. Samples were centrifuged at 30,000×g (15,000 rpm, JA–17 rotor) for 20 min, and then the supernatant was transferred to vials for VFA analysis. The VFAs were estimated by gas chromatography (GC Thermo TRACE 1300) using capillary column (TR-FFAP 30 m × 0.53 mmI D × 0.5 μm) film (thermo-part NO: 260 N225 P) and the temperature was increased from 100 to 200 °C at a rate of 10 °C/min. The injection and flame ionization detector (FID) temperature was set at 220 °C and 250 °C, respectively. The carrier gas nitrogen was set at a 7 ml/min flow rate, gas flow air at 450 ml/min, hydrogen at 40 mL/min, and make-up gas at 35 mL /min. A mixture of VFA of known concentrations was used as a standard for calibration. For Lactic acid determination, 1 ml of liquid phase was taken into 1.5 ml Eppendorf tube and centrifuged at 30,000×g (15,000 rpm, JA–17rotor) for 20 min at 4 °C. Lactic acid was determined colorimetrically as described in the protocol of Borshchevskaya, et al. [41].

### Experiment 3

The effect of CIN and MON supplementation on in vitro fermentation of barley and corn grains was investigated. Barley and corn grains were grounded to pass a 1 mm screen and used as substrates. About 4.5 g of ground grains in triplicates were placed into serum bottles to induce rumen acidosis, according to Dennis, et al. [42]. The incubation medium, the dose of MON and CIN, and sample collection were the same as described in Exp. 2. The experiment includes 6 treatments as follows: 2 substrates (barley and corn grains) and each substrate fortified with control (without any supplementation), CIN, and MON.

### Statistical analyses

The data was statistically analyzed using the MIXED procedure of SAS (version 9.1, SAS Inst., INC., Cary, NC). In EXP 1, the following model was assumed: Y_ij_ = μ + T_i_ + e_ij_ where: μ is the overall mean, T_i_ is the treatment, e_ij_ is the random error term. Data of EXP. 2 were statistically analyzed by factorial arrangement using the following Model 1: Y_ijk_ = μ + E_i_ + T_j_ + ET_ij_ + e_ijk_, where Y_ijk_ = the measured parameter, μ = the overall mean, E_i_ = the main effects of treatment (control, CIN, and MON) i^th^ treatment, T_j_ = the main effect of j^th^ incubation time, ET_ij_ = the interaction between the i^th^ treatment and incubation time and eijkl = random error. Data of EXP. 3 were analyzed as in EXP. 2 to identify the main effects of treatment, substrate (corn and barley), and incubation time were included in the model with the correct interactions. The differences between treatments were considered significant at *P* < 0.05. The Tukey’s multiple range post hoc test was used for pairwise comparisons.

## Results

### Experiment 1

**Fig. 1:**
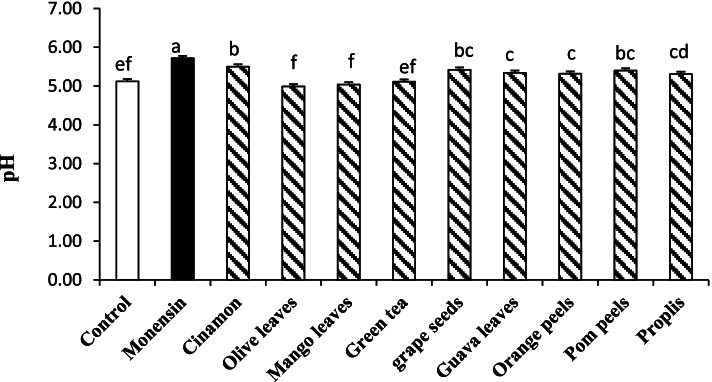
Effect of monensin (12 μg/ml) and plant extracts (1.5 mg/ml) addition to rumen fluid (in vitro) on ruminal pH up to 6 h incubation using hay (0.01 g/ml rumen fluid) and D-glucose (0.1 g/ml rumen fluid) as a substrate (EXP.1). The treatments were control (without supplementation), monensin, pomegranate peels extract**,** orange peels extract, grape seed extract, olive leaf extract, guava leaf extract, mango leaf extract, green tea leaf extract, propolis extract, and cinnamon extract. ^a-c^ Means in the column with different superscripts differ (*P* < 0.001). SEM =0.06

As presented in Fig. 1, there was wide variation among plant extracts in their effect on pH. Initially, compared to control, all tested plant extracts significantly (*P* < 0.05) increased the pH values except mango, green tea, and olive extracts. The maximum increment in pH was recorded in CIN and MON. The effect of other plant extracts on pH was as follows: pomegranate peels and grape seeds extracts > guava and orange peel extracts > propolis extract.

**Fig. 2:**
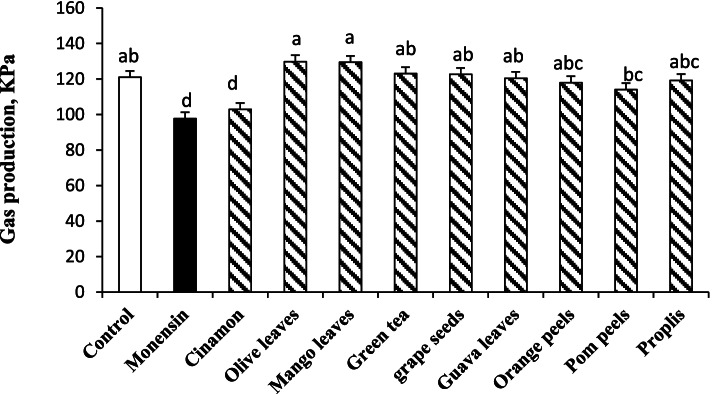
Effect of monensin (12 μg/ml) and plant extracts (1.5 mg/ml) addition to rumen fluid (in vitro) on cumulative gas production (KPa) over 6 h incubation using hay (0.01 g/ml rumen fluid) and D-glucose (0.1 g/ml rumen fluid) as a substrate (EXP1). The treatments were control (without supplementation), monensin, pomegranate peels extract**,** orange peels extract, grape seed extract, olive leaf extract, guava leaf extract, mango leaf extract, green tea leaf extract, propolis extract, and cinnamon extract. SEM =3.6; ^a-c^ Means in the column with different superscripts differ (*P* < 0.001)

As shown in Fig. 2, the lowest rate of cumulative gas production (*P* < 0.01) was observed with MON (97.69 kPa) and cinnamon (102.02 kPa) compared to control (120.98 kPa). On the other hand, no significant differences were observed with other plant extracts on cumulative gas production compared to control.

### Experiment 2

Effect of treatment, time, and treatment × time interaction (*P* < 0.001) was significant with ruminal pH. The addition of CIN significantly increased (*P* < 0.01) ruminal pH (6.14) compared to CON (5.88) at the first 6 h of incubation. However, there were no significant differences in pH values between CIN and CON at 12 and 24 h of incubation. On the other hand, ruminal pH was higher (*P* < 0.01) with MON than CON and CIN in all incubation times (Table 2).

**Table 2:** Effect of monensin (12 μg/ml) and cinnamon extract (1.5 mg/ml) addition on ruminal pH and cumulative gas production up to 24 h of incubation period using glucose as a substrate (Exp. 2)

Items	Treatments			
	Control	Monensin	Cinnamon extract	SEM^**1**^
**pH**				
6 hours	5.88 ^c^	6.48 ^a^	6.14 ^b^	0.042
12 hours	4.53 ^b^	5.04 ^a^	4.43 ^b^	0.022
24 hours	4.14 ^b^	4.51 ^a^	4.17 ^b^	0.024
**Cumulative gas production (KPa)**				
3 hours	57.12 ^a^	40.31 ^b^	50.33 ^a^	1.65
6 hours	124.39 ^a^	81.38 ^c^	104.81 ^b^	7.05
9 hours	146.69 ^a^	111.79 ^b^	140.89^a^	6.27
12 hours	160.17 ^a^	133.94 ^b^	154.03^a^	5.31
24 hours	173.03 ^a^	154.70 ^b^	164.79^a^	4.42

^a-c^ Means in the same row with different superscripts differ (*P* < 0.05)

^1^*S.E.M* Standard error of the mean. The treatments were control (without any supplementation), cinnamon extract, and monensin

The *p*-value for the effect of treatment, time, and interaction was < 0.001 for pH and cumulative gas production

Also, there was a significant (*P* < 0.001) effect for treatments, time, and their interaction on cumulative gas production (Table 2). No significant differences in cumulative gas production were observed between CON and CIN during incubation times except at 6 h where CIN significantly (*P* < 0.01) reduced cumulative gas production. On the contrary, MON significantly decreased (*P* < 0.01) cumulative gas production in all incubation times compared to CON and CIN.

As demonstrated in Table 3, the concentration of lactic acid was lower (*P* < 0.01) in MON (45.60 mM) than CIN (70.64 mM) or CON (86.80 mM). Both CIN and MON decreased (*P* < 0.01) total VFA concentration (83.89 mM and 65.73 mM) compared to control (100.25 mM) after 24 h of incubation. The addition of CIN increased the molar concentration of acetate and reduced the molar concentration of propionate (*P* < 0.01) compared to CON and MON. Consequently, the highest value of acetate: propionate ratio (4.17) was found with CIN compared to CON (3.75) and MON (3.46).

**Table 3:** Effect of monensin (12 μg/ml) and cinnamon extract (1.5 mg/ml) addition on rumen fermentation products (in-vitro) over an incubation period of 24 h using hay and glucose as a substrate (Exp 2)

Items	Control	Monensin	cinnamon	SEM^**1**^	***P***-value
Lactic acid (mM)	86.8^a^	45.60^c^	70.67^b^	3.00	< 0.001
Total VFA (mM)^2^	100.25^a^	65.73^c^	83.89^b^	2.26	< 0.001
Acetate, %	68.84^b^	67.84^c^	70.43^a^	0.3	< 0.001
Propionate, %	18.37^b^	19.63^a^	16.89^c^	0.2	< 0.001
Butyrate, %	10.63	10.71	10.82	0.17	0.741
Acetate: Propionate	3.75^b^	3.46^c^	4.17^a^	0.054	< 0.001

^a-c^ Means in the same row with different superscripts differ (*P* < 0.001)

^1^*S.E.M* Standard error of the mean

^2^*VFA* Total volatile fatty acids

### Experiment 3

**Fig. 3:**
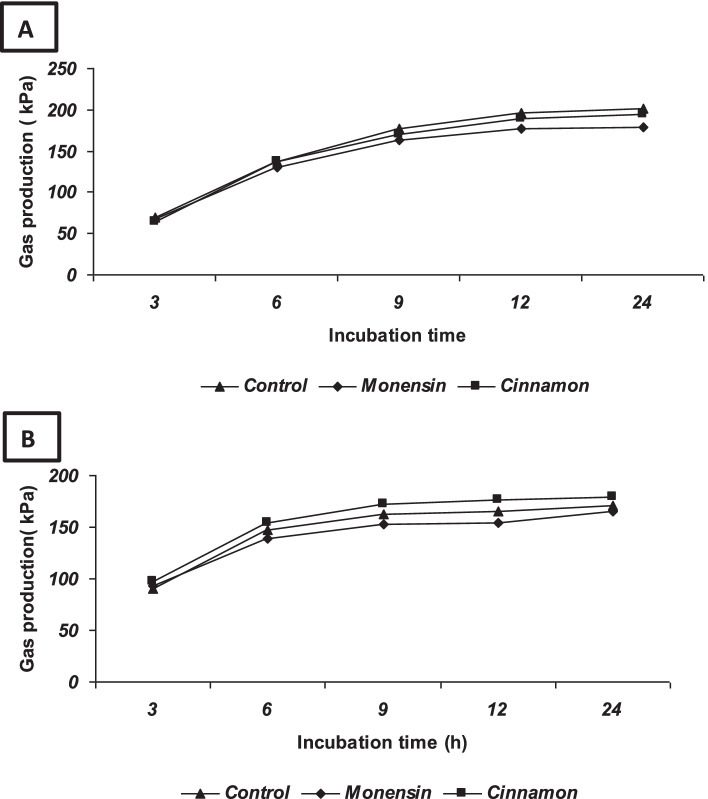
Effect of monensin (12 μg/ml) and cinnamon extract (1.5 mg/ml) addition on cumulative gas production (KPa) from in-vitro fermentation over 24 h incubation period (3, 6, 9, 12, and 24 h) using corn (A) or barley grain (B) as substrates (Exp.3). Symbols represent means, *n* = 6. The p-value for the effect of time, substrate x time, and treatment x time was < 0.001, while =0.09 for the treatment effect. The p-value for substrate effect was 0.56, substrate x treatment =0.39, and substrate x treatments x time = 0.08. SEM: standard error of the mean 4.87

Effects of MON and CIN addition on cumulative gas production (kPa) using corn or barley as substrate are shown in Fig. 3**.** No significant (*P* > 0.05) differences were observed in the effect of treatment and substrate on cumulative gas production. No interactions were observed between treatment and substrate. On the contrary, a significant (*P* < 0.001) interaction between treatment × time and substrate × time was observed.

Changes in ruminal pH values with corn and barley as substrates at different incubation times are presented in Table 4. An interaction (*P* < 0.001) between substrate and time on pH value was observed. There was a significant effect of substrate (*P* < 0.001) on ruminal pH where barley reduced overall ruminal pH (4.64) compared to corn (4.98). Also, an interaction (*P* < 0.01) was observed between treatment × substrate × time. When corn was used as a substrate, the addition of CIN and MON significantly increased (*P* < 0.05) ruminal pH compared to control at 6 h and 24 h of incubation; however, no effect of MON and CIN on pH values at 12 h compared to CON. On the other hand, when barely used as a substrate, MON did not affect pH at 6 and 12 h; however, it increased (*P* < 0.01) ruminal pH at 24 h compared to CIN and CON.

**Table 4:** Effect of monensin (12 μg/ml) and cinnamon extract (1.5 mg/ml) addition on ruminal pH at different incubation times using corn and barley grains as substrates (Exp. 3)

Substrates:	Corn			Barley		
Treatments:	Control	Monensin	Cinnamon extract	Control	Monensin	Cinnamon extract
Time (h)						
6	5.37^Ab^	5.61^Aa^	5.54^Aa^	5.13^A^	5.13^A^	5.10^A^
12	4.98^B^	4.98^B^	5.03^B^	4.56^B^	4.63^B^	4.59^B^
24	4.35^Cb^	4.46^Ca^	4.50^Ca^	4.16^Cb^	4.30^Ca^	4.18^Cb^
Overall	4.98			4.64		
SEM	0.034					

^a-b^ Means in the same row with different superscripts differ within substrate (*P* < 0.001)

^A-C^ Means in the same column with different superscripts differ (*P* < 0.001)

*SEM* Standard error of the mean

The *P*-Value for substrate effect was < 0.001, for treatment effect was < 0.05; for the interaction effect between substrate and time was < 0.001, and for the interaction effect between the substrate, treatment, and time was < 0.01. The interaction effect between substrate and treatment was not statistically significant

The effects of MON and CIN on rumen fermentation products at 24 h incubation time using corn and barley as substrates are presented in Table 5. A significant (*P* < 0.01) interaction was found between substrate and treatment, resulting in reduced lactic acid concentration for CIN treatment compared to CON and MON when corn was used as a substrate. The effect of the substrate was significant (*P* < 0.01) as barley caused a higher lactic acid and acetate production compared to corn. However, no effect of the substrate was found on propionic acid and total VFA. MON increased (*P* < 0.01) propionic acid and reduced acetate concentrations compared to CON and CIN with both substrates (corn and barley), resulting in lower acetate: propionate ratio for MON compared to CON and CIN with both substrates. Also, MON significantly reduced (*P* < 0.01) butyrate concentration compared to CIN and CON with both corn and barley as substrates.

**Table 5:** Effect of monensin (12 μg/ml) and cinnamon extract (1.5 mg/ml) addition on rumen fermentation products at 24 h using corn and barley grains as substrate (Exp. 3)

Items	Corn			Barely			SEM^**2**^
	Treatments^**1**^						
	CON	MON	CIN	CON	MON	CIN	
Lactic acid (mM)	38.30^c^	28.09^cd^	21.07^d^	139.79^a^	103.41^b^	141.7^a^	4.17
Total VFA (mM)	218.1	209.9	214.9	205.3	220.4	189.6	7.4
Acetate %	58.09^b^	53.36^c^	58.62^b^	61.74^a^	58.20^b^	60.86^a^	0.4
Propionate %	23.1^d^	31.6^a^	24.1^cd^	24.7^cd^	29.8^b^	24.8^c^	0.4
Butyrate%	16.5^a^	12.96^c^	15.2^b^	12.4^d^	10.6^e^	12.7^cd^	0.1
Acetate: Propionate	2.52 ^a^	1.69^b^	2.43^a^	2.57^a^	1.95^b^	2.45^a^	0.05

^a-d^ Means in the same row with different superscripts differ (*P* < 0.001)

^1^*CON* Control, *MON* Monensin, *CIN* Cinnamon extract

^2^SEM* Standard error of the mean, SEM for monensin was 6.1, 0.3, 0.04 for lactic acid, propionic, and acetate: propionate, respectively

The p-value of the substrate and treatment effects was < 0.01 for lactic acid, acetate, butyrate, and acetate: propionate ratio. The *p*-value of the treatment effect was < 0.01, but no significant effect of substrate on propionate. There was no significant effect (*P* > 0.05) of substrate and treatments on TVFA. The p-value of the substrate x treatment interaction was < 0.01 for lactic acid, propionate, and butyrate. No significant effect (*P* > 0.05) of the substrate x treatment interaction on acetic acid, TVFA, and acetate: propionate ratio

## Discussion

Nine plant extracts were used to examine their efficiency in preventing acidosis conditions. In Exp.1, an in-vitro bioassay [21] was used to invoke an acidosis environment with glucose used at a high concentration (4.5 g/45Ml) as the main substrate and the fermentation time was only 6 h. The assay effectively simulated acidosis conditions as it lowered the pH of control tubes to 5.1. MON was the most effective treatment in controlling pH; however, it reduced accumulated gas production. The mode of action of MON is related to its ability to prevent the accumulation of lactic acid through its selective effect against lactate-producing bacteria (*streptococcus bovis*) [43]. The inhibition of *streptococcus bovis* prevented pH decline and stimulated lactic acid fermenting bacteria like *Megasphera elsdenii* [22].

The plant extracts used in this screening bioassay were chosen for their higher content of phenolic compounds. Xia, et al. [15] and Salem, et al. [44] reported that plant extracts with a high phenolic compound concentration have potent antimicrobial activity against lactate-producing bacteria. In this experiment, the addition of grape seed, guava leaves, orange peels, pomegranate peels, propolis, and CIN maintained higher pH compared with the control. This indicates that these plant extracts have antimicrobial activity against lactate-producing bacteria while others (olive, mango, and green tea leaves) did not exert antimicrobial activity. The variation between different plant extracts in their effect on lactate-producing bacteria could be related to the differences in the types and concentrations of active components [23]. Despite their high contents of TPh (336.52 mg/ g DM) and TF (3.60 mg/ g DM), Grape seeds were not as effective as CIN in increasing ruminal pH. This could be due to the possibility of an antagonistic effect between some phenolic compounds and/or flavonoids present in grape seed extract [45].

In this study, CIN efficiently increased pH more than the other plant extracts. Cinnamon is derived from a Greek word that means sweet wood obtained from the inner bark of trees genus *Cinnamomum* and belonging to *Lauraceae* family [46]. The efficiency of CIN in maintaining pH could be attributed to its higher concentration of condensed Tannins (CTS) (28.06 g/g DM). Condensed tannin is formed by polymers of (flavan-3-ol) units bound by carbon-carbon bonds [47]. Therefore, rumen microbes cannot degrade the carbon-carbon bonds of condensed tannins [48, 49]. Jones, et al. [50] reported that condensed tannins of Sainfoin *(Onobrychris viciifolia)* decreased the growth of several ruminal bacteria such as *Butyrivibrio fibrisolvens, Streptococcus bovis, Prevotella ruminicola,* and *Ruminobacter amylophilis*. Such inhibitory effects are due to the binding of condensed tannins to the bacterial cell wall and deactivating the cell-bound extracellular enzymes [50]. Besides, the antimicrobial activity of CIN could be possibly due to its high content of cinnamaldehyde which was not estimated in this study. Cinnamaldehyde is considered the main bioactive in CIN [51]. Cinnamaldehyde’s antimicrobial effect is not well understood, but it may be related to its ability to interact with bacterial proteins deeper in the cell. It did not affect cell membrane stability like other secondary plant metabolites [52].

In EXP.1, CIN achieved the most favorable effect on ruminal acidosis. Hence, it was chosen to be evaluated either with glucose (EXP.2) or grain substrates (EXP.3). In EXP. 2, CIN maintained greater pH than control due to its high content of antimicrobial compounds against lactic acid-producing bacteria. However, this action could not be sustained up to 12 and 24 h of incubation. When glucose was used as a substrate (EXP. 2), the MON addition increased the pH and decreased lactic acid concentrations compared to CON up to 24 h of incubation. But, MON could not raise the pH above 5.0, indicating that the protection assay was truly effective against sub-acute acidosis up to 6 h for CIN and 12 h for MON. Furthermore, a similar trend was obtained when glucose was replaced by corn or barely (EXP.3). This could be attributed to the higher concentration of substrates (glucose, EXP.2) and grains (corn or barely, EXP.3) that were used (4.5 g/45 ml) to induce acidosis.

Under normal fermentation conditions, lactic acid is an intermediate product in the rumen in carbohydrate fermentation that is readily converted into VFA by lactate-consuming bacteria [21]. However, the consumption of high concentrates with highly fermentable carbohydrates leads to the accumulation of lactic acid, which is more vital than VFA, causing a rapid decline in pH and consequently reducing the activity of lactate-fermenting bacteria especially when pH declines below 5.5 [43]. The drop of pH is more rapid if lactic acid concentration reaches 20 mM as the pH becomes less than 5.0, and the lactate-producing bacteria will become the dominant bacteria [43].

The total VFA concentration was not affected by CIN when corn or barely was used as substrate (EXP. 3), indicating that CIN maintained balanced microbial activity. However, CIN reduced total VFA concentration when glucose was the substrate (EXP. 2). The inconsistent effect of CIN in EXP. 2 and 3 on total VFA is not understood. This effect can be attributed to the difference in the substrate used and the experimental conditions. The finding that CIN increased the acetate/propionate ratio in EXP. 2 was contrary to MON, which reduced the acetate/propionate ratio. The selective effect of MON against lactate-producing bacteria enhanced the activity of lactate-fermenting bacteria such as *Megasphera elsdenii* that convert lactic acid to propionate, thus lowering the acetate to propionate ratio [43]. Cardozo, et al. [53] reported that the effect of CIN extract and cinnamaldehyde on acetate/propionate ratio in-vitro was pH-dependent during fermentation. They reported that when the pH at the start of fermentation was 7.0, the acetate to propionate ratio increased, and the acetate to propionate ratio decreased when the pH was 5.5. The pH values remained unchanged throughout their fermentation time. The pH values were decreased markedly during fermentation time in our study because we used a larger amount of substrate (4.5 g/45 ml) to induce acidosis, according to Dennis, et al. [42], compared to the smaller amount of substrate (0.5 g /50 ml) used by Cardozo, et al. [53]. Also, Cardozo, et al. [53] reported that the higher concentration of CIN caused an increased acetate to propionate ratio. In the present EXP., the CIN was used at a 1.5 mg/ml concentration, according to Busquet et al. (2006). They reported that a plant extract concentration exceeding 3 mg/ml could cause microbial inhibition. In the preliminary experiment (data are not shown), we used a 1 mg/ ml concentration for all plant extract used in EXP.1, but no significant differences were observed.

Another explanation for the greater acetate concentration In EXP. 2 is that the tannins present in CIN could stimulate bacteria to produce acetate from glucose. Zhao, et al. [54] reported that tannins simulated the growth of acetate-producing bacteria such as Rikenellaceae RC9, which produce acetate from glucose. This observation of higher acetate production with the addition of CIN was obvious when glucose was the substrate (EXP. 2), indicating the varying effect of substrate (glucose vs. corn/ barely) on acetate concentration. Thus, the effect of CIN on acetate to propionate ratio depends on pH, dose, substrate, and experimental conditions [53].

As expected, barley reduced rumen pH value and increased lactic acid concentration compared to corn, which agrees with Fulton et al. (1979), who reported that steers fed wheat as concentrate had lower ruminal pH than steers fed a corn-based diet. Despite having more starch than barley, corn’s starch is less available for degradation by rumen bacteria due to the less degradable protein membrane surrounding its starch granules [55]. This indicates the importance of grain type on ruminal pH and hence acidosis [56]. The ability of CIN to increase rumen pH when corn was used as a substrate compared to barely indicates that the antimicrobial effect of CIN becomes less observable when the degradation of starch is very high as in barely, leading to a higher microbial population that may require a higher dose of CIN to inhibit lactate-producing bacteria.

## Conclusion

Compared to other plant extracts, CIN was the best plant extract after MON to prevent rumen acidosis in-vitro. Also, cinnamon altered ruminal microbial fermentation by increasing the acetate to propionate ratio. This may indicate that CIN is a beneficial additive to transition diets for lactating animals. Cinnamon had beneficial effects when corn was used as a substrate on rumen fermentation profiles by increasing ruminal pH and decreasing lactic acid concentration. Further investigations are required in-vitro and in-vivo to study the impact of CIN on controlling ruminal acidosis.

## Data Availability

The datasets used and/or analyzed during the current study are available from the corresponding author on reasonable request.
